# BRCA1 expression, proliferative and apoptotic activities in ovarian epithelial inclusions

**DOI:** 10.1186/s13048-017-0307-6

**Published:** 2017-03-07

**Authors:** Yiying Wang, Yue Wang, Li Wei, Shuhui Hong, Miaoqing Zhao, Xi Zhang, Wenxin Zheng

**Affiliations:** 1 Department of Obstetrics and Gynecology, Henan Province People’s Hospital, Zhengzhou, China; 2 Department of Obstetrics and Gynecology, Zhengzhou University People’s Hospital, Zhengzhou, China; 3 Department of Gynecology, Qianfoshan Hospital of Shandong University, Ji’nan, China; 4 Department of Pathology, Provincial Hospital Affiliated to Shandong University, Ji’nan, China; 5 Department of Obstetrics and Gynecology, Qilu Hospital of Shandong University, Ji’nan, China; 6 Department of Pathology, University of Texas Southwestern Medical Center, 6000 Harry Hines Blvd, NB6.408, Dallas, 75390-9072 TX USA; 7 Department of Obstetrics and Gynecology, University of Texas Southwestern Medical Center, 6000 Harry Hines Blvd, NB6.408, Dallas, 75390-9072 TX USA; 8 Harold C. Simmons Comprehensive Cancer Center, University of Texas Southwestern Medical Center, Dallas, TX USA

**Keywords:** Ovarian cancer precursors, Ovarian epithelial inclusions, BRCA1, Ovarian epithelial proliferation and apoptosis

## Abstract

**Background:**

The purpose of this study was to examine proliferative and apoptotic activity in relation with BRCA1 expression in ovarian epithelial inclusions (OEIs), the putative precursor lesions of ovarian epithelial cancer (OEC).

**Methods:**

Benign ovaries from 95 patients were examined. Dual immunohistochemical staining for both BRCA1 and MIB-1 were performed to examine the relationship between BRCA1 and MIB-1 in OEI cells. Apoptotic activity was assessed on the parallel tissue sections by using TUNEL assay. Patients’ age, menstrual phase and menopausal status were analyzed.

**Results:**

OEIs were present in the ovaries of 53% of the patients. OEIs were less frequently found in premenopausal (45%) than postmenopausal women (58%). BRCA1 and MIB-1 were found in 27 and 47% of the OEI-containing ovaries, respectively. All BRCA1 positive OEI cells are MIB-1 positive with dual staining method, although overall the percentage of positive cells was small. No significant difference was found for BRCA1 and MIB-1 expression in OEIs between menopausal status and menstrual phases. Apoptosis containing OEIs were seen in 70% of the ovaries. Compared to OEIs in proliferative menstrual phase and premenopausal status, significantly more apoptosis was found in OEIs from secretory phase and postmenopausal women. A small fraction of the epithelial cells within OEIs are proliferating or dying.

**Conclusions:**

Low estrogen and/or high progesterone levels may promote OEI cell turnover via induction of apoptosis. Imbalance between cell proliferation and death within OEIs under influence of hormones may play a role in the ovarian epithelial tumorigenesis.

## Background

Epidemiological studies show a clear trend of significantly decreasing risk of ovarian epithelial cancer (OEC) with increasing numbers of pregnancies, duration of oral contraceptive [1] use, and duration of breast-feeding [2]. These protective factors interrupt ovulation and associated cyclic hormonal changes, but the molecular mechanisms accounting for the protective effects are unknown. Hypotheses that have been suggested to explain these observations from different angles including ‘incessant ovulation’ and ‘imbalanced levels of gonadotropins and sex-steroid hormones’ [3].

The incessant ovulation hypothesis put forward by Fathalla [4] proposes that OEC risk is essentially determined by the increased proliferative activity of the ovarian surface epithelium (OSE) required to accomplish repair of the ovarian surface after each ovulation. It is supported by experiments in which repeated culture of rat ovarian epithelial cells resulted in their acquiring malignant features and the production of serous adenocarcinomas when injected into nude mice [5–7]. The incessant ovulation hypothesis provides a quantitative explanation of the decreasing risk with pregnancies, OC use and breast feeding [8]. But it cannot explain the continuing increase in OEC rates after menopause or the much reduced incidence rates of OEC in Asian women [8].

A simple incessant ovulation hypothesis also does not provide a satisfactory explanation for the observation that most early stage OECs are found within the ovary rather than on its surface [9]. This observation has led most investigators to conclude that OECs arise principally from ovarian epithelial inclusions (OEIs) rather than directly from the OSE [9], but additional evidence for this hypothesis has been difficult to produce. Mittal et al., [10] studied contralateral ovaries from 42 women with unilateral ovarian cancer and compared them to ovaries from age-matched controls. They reasoned that if OEC did arise from OEI, then these contralateral ovaries from OEC patients should have increased numbers of OEIs, and they actually found this in their study. Presence of OEI is also associated with the development of OEC [11]. However, three other similar studies failed to confirm this [12–14]. Increased numbers of OEI have been found in ovaries from women with a strong familial history of ovarian cancer than in ovaries from control women in two studies [15, 16]. It is, however, difficult to judge the significance of these results as these studies also found multiple other ‘abnormalities’ in the ovaries of the women with a family history of ovarian cancer. More recently, understanding the cell of origin of OECs, particularly ovarian serous carcinomas, have been significantly improved. It is currently believed that the majority of ovarian serous carcinomas are derived from fallopian tubal epithelial cells [17, 18].

OEIs were previously thought to arise from entrapment of the ovarian surface after ovulation, but our recent studies showed that the majority OEIs are actually derived from fallopian tubes [17, 19–21] . The OEIs from fallopian tube is also known as endosalpingiosis. From that perspective, OEIs or endosalpingiosis are now considered as precursor lesions of OECs. It may, therefore, be more relevant to study the proliferative and apoptotic rate of OEI cells than the number of OEIs, since there is much evidence that cell proliferation and apoptosis are strongly associated with cancer development and progression [22]. If OEI cells do give rise to OECs, it will be then very reasonable to hypothesize that epithelial proliferation is increased and/or apoptosis is reduced in OEIs in the postmenopausal compared to the premenopausal period since there is a much slower increase in ovarian cancer risk in the postmenopausal period [8].

Women who carry BRCA1 mutations are at a significantly increased risk for ovarian cancer [1] and high prevalence of OEIs have been found in prophylactically removed ovaries of BRCA mutation carriers [23]. BRCA1 behaves like a tumor suppressor gene with DNA repair function [24]. If OEIs are the site of OEC development then dividing OEI cells may express BRCA1 to exert its protective effect against the development of OEC. We previously reported that BRCA1 expression paralleled cell proliferation in benign and borderline ovarian epithelial tumors, but not in ovarian epithelial cancers [25]. Sporadic ovarian cancer showed significantly reduced levels, rather than complete loss, of BRCA1 expression [25]. More interestingly, OEI or endosalpingiosis has also been linked to the development of pelvic serous carcinoma in women who are BRCA mutation carriers in one of our recent studies [26].

In this paper, we describe our findings on the occurrence of OEIs, and on cell proliferation, cell apoptosis, and BRCA1 expression of OEI epithelial cells in ‘normal’ ovaries from women with no known history of familial ovarian cancer. Age, menopausal status, and menstrual phase were considered in the analysis.

## Methods

### Patients and tissue sections

‘Normal’ ovaries from 95 patients of known menopausal status were obtained from the files of the Department of Pathology, University of Arizona. A total of 190 ovaries were obtained at total abdominal hysterectomy and bilateral salpingo-oophorectomy for benign conditions (but excluding benign ovarian tumors) during the period 2010 through 2015. Patients studied were without a known family history of breast or ovarian cancers. Approval for use of these tissues was obtained from the Institutional Review Board of the University of Arizona.

Two sections or more from each ovary were examined for the presence of OEIs. If both ovaries from a single patient contained OEIs, we arbitrarily chose one of the ovaries for the study. The number of OEIs in each section was recorded separately. Although ovaries from 50 patients showed the presence of OEIs, we studied MIB-1 and BRCA1 expression in ovaries of 30 patients due to limited tissue availability.

Menstrual phase was determined based on microscopic examination of the endometrium from hysterectomy specimens. Menopausal status was obtained from clinical charts.

### Immunohistochemical (IHC) analyses and dual stainings

Representative sections containing OEIs were stained with dual markers of BRCA1 and MIB-1. Mouse monoclonal antibody against human BRCA1 protein (Ab-1, IgG2a) generated from amino acid sequence 1–304 of BRCA1 was obtained from Oncogene Research Products (Cambridge, MA). The specificity of this BRCA1 antibody has been characterized previously [25]. MIB-1 (Ki-67 paraffin), a mouse monoclonal antibody (IgG1) recognizing a nuclear antigen expressed in all cell cycle phase except G_0_ [27], was obtained from Immunotech, Inc. (Westbrook, ME). Five-micrometer parallel sections of the ovaries containing OEIs were cut and placed on Super Plus slides (Fisher Scientific, Pittsburgh, PA) for IHC. A section of each specimen was stained with hematoxylin and eosin (H&E) and examined microscopically for the presence of OEI and to confirm that the ovarian specimen was otherwise benign. The dual staining procedure was described elsewhere [28]. Briefly, the first (BRCA1) IHC staining was performed on formalin-fixed and paraffin-embedded sections using the streptavidin-biotin-peroxidase methodology and the second (MIB-1) was done with alkaline phosphatase. The antigens were unmasked with the heat-mediated antigen retrieval method as described previously [25]. Specific signal of BRCA1 was visualized by incubation with peroxidase coupled rabbit anti-mouse IgG for 120 min, followed by incubation with Diaminobenzidine as chromogen creating a brown nuclear staining product. Then MIB-1 IHC was performed as described previously [25] by using alkaline phosphatase coupled secondary antibodies. Subsequent color development with phosphate substrate created a bright red product. An orange color was observed when both BRCA1 and MIB-1 stained the same cell nuclei (double nuclear staining). Five hundred OEI epithelial cells per ovary were counted to generate the percentage of positive cells.

Controls for the IHC were as follows: MCF-7 breast cancer cells and the developing follicles in human ovarian sections served as positive controls for BRCA1 expression [29]. Internal positive controls were evaluated by utilizing areas of folliculogenesis when ovaries were from premenopausal women. Ab1 BRCA1 antibody was also validated in two ovarian cancers containing germline BRCA1 del185AG mutations. This specific mutation results in the deletion of most of the protein, including the Ab-1 epitope. As expected, no BRCA1 immunoreactivity was detected in these two cases. Cytokeratin IHC was done in all BRCA1 negative cases to rule out false negatives. Proliferative endometrial tissue sections served as positive controls for MIB-1 staining. Negative controls were carried out by replacing primary antibodies with class matched mouse IgGs on parallel ovarian sections.

### Detection of apoptosis

Parallel sections from the above mentioned 30 ovaries were examined for apoptosis by using terminal deoxynucleotidyl transferase-mediated deoxyuridine triphosphate nick end labeling (TUNEL) assay. The TUNEL procedure was previously described [30]. The ApopTag In situ Apoptosis Detection Kit (Oncor, Gaithersburg, MD) was used and the procedure protocol recommended by the manufacturer was followed. Briefly, deparaffinized and hydrated slides were treated with proteinase K (20 μg/ml) for 15 min at room temperature. Slides were thoroughly washed in PBS then treated with 2% hydrogen peroxide for 5 min to bleach the endogenous peroxidase activity. Samples were then subjected to 3’-end labeling of DNA with digoxigenin-dUTP utilizing TdT enzyme in a humidifier chamber at 37 °C for 60 min. This was followed by incubation with conjugated anti-digoxigenin peroxidase antibody. Sections were developed with diaminobenzidine tetrahydrochloride substrate, counter-stained, dehydrated, cleared and mounted. Apoptotic cells were assessed in the epithelial compartment only under light microscopic examination. Apoptotic cells were counted based on their golden brown stained, condensed, lobulated, and fragmented nuclei. A total of 500 cells were counted in high power fields (400×) and the percentage of apoptotic cells was determined.

### Data evaluation and statistical analysis

Ovaries with or without positively stained cells for each assay (MIB-1, BRCA1 and TUNEL) were calculated and recorded. The number of OEIs from each ovary was also recorded. A total of 500 cells were counted in high power fields (400×) and the percentage of positively stained (MIB-1 and BRCA1) and apoptotic cells was determined by counting the OEI cells in the stained sections in a random fashion. Based on the obtained data, the average percentage of positive cells detected by the above mentioned assays in each OEI was calculated and compared in different age groups.

The data were analyzed by standard contingency table methods and non-parametric Mann-Whitney U-tests using the EPILOG (Epicenter Software, Pasadena, CA) and StatView (SAS Institute, Cary, NC) computer package programs. Fisher’s exact tests were used to calculate two-side *p* values.

## Results

### Presence of OEIs

OEIs were present (in the two sections examined) in the ovaries of 50 (53%) of the 95 patients with an average of 0.99 inclusions (range 0–13) per ovarian section examined. The presence of OEIs (from the sum of 2 sections) in the paired ovaries were highly correlated, 44 (88%) of the 50 cases with OEIs had OEIs in both ovaries. OEIs were found slightly less frequently in ovaries from premenopausal women (18/40, 45%) than in ovaries from postmenopausal women (32/55, 58%) (Table 1); the average numbers of OEIs per ovarian section examined were 0.65 and 1.25 in premenopausal and postmenopausal women, respectively. There was no clear trend with age in premenopausal women. In premenopausal women in the age groups 20–34, 35–44, and 45–54, the presence of OEIs were: 4/6 (67%), 3/12 (25%) and 11/22 (50%), respectively. The average numbers of OEIs per ovarian section examined for each age group were 1.08, 0.25 and 0.75, respectively. In postmenopausal women there was a much greater number of OEIs in the age group 45–54 than in older groups. In the age groups 45–54, 55–64, and 65–79, the presence of OEI was 14/18 (78%), 11/21 (52%), and 7/16 (44%), respectively. The average number of OEIs per ovarian section examined was 2.25, 0.89 and 0.58, respectively. The average number decreased significantly with age among postmenopausal women (*p* = 0.001) from age group 45–54 to 65–79.

**Table 1:**
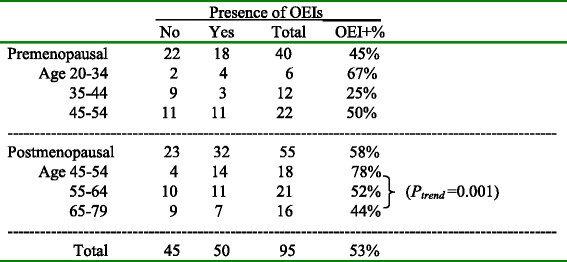
Presence of OEIs in ovarian sections of pre- and postmenopausal women

### MIB-1 and BRCA1 expression in OEIs

We examined MIB-1 immunoreactivity in OEI cells from 30 OEI-containing ovaries, among which MIB-1 was found in OEI cells from 14 (47%) ovaries. Premenopausal OEI-containing ovaries showed greater proportion of positive immunoreactivity (7/13, 54%) than postmenopausal ovaries (7/17, 41%) (Table 2). However, the average percentage of stained cells was slightly greater in the postmenopausal (0.76%) than in the premenopausal OEIs (0.69%). The MIB-1 immunoreactivity was present more often in the proliferative phase (5/8, 63%) than in the secretory phase (2/5, 40%; Table 2), and the average percentage of stained cells was 0.88 and 0.40%, respectively. All these differences were, however, based on small numbers and were statistically insignificant.

**Table 2:** IHC examination of MIB-1 and BRCA1 expression in OEIs of certain menopausal status and menstrual cycle phase

	MIB-1		BRCA1	
	Negative	Positive (%)	Negative	Positive (%)
Premenopausal	6	7 (54%)	9	4 (31%)
Postmenopausal	10	7 (41%)	13	4 (24%)
Total	16	14 (47%)	22	8 (27%)
Proliferative phase	3	5 (63%)	5	3 (38%)
Secretory phase	3	2 (40%)	4	1 (20%)
Total	6	7 (54%)	9	4 (31%)

**Fig. 1:**
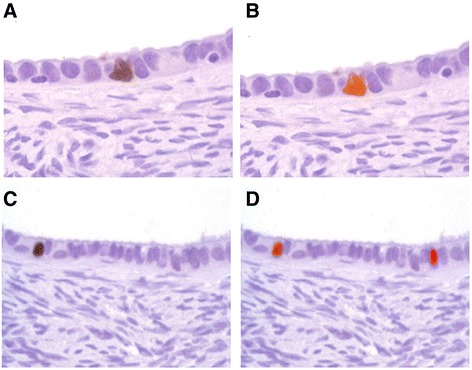
BRCA1 and MIB-1 dual immunohistochemical stainings in ovarian epithelial inclusions. One epithelial cell of each representative OEIs showed nuclear immunoreactivity of BRCA1 (**a** and **c**, *brown in color*). These two BRCA1 positive cells were also stained by MIB-1 (**b** and **d**, *orange in color*). Additional one MIB-1 positive cell was seen (**d**, *red in color on the right side*). No cytoplasmic or stromal positivity is seen. (Original magnifications × 200)

BRCA1 immunoreactivity was found in OEI cells from 8 (27%) of the 30 OEI-containing ovaries examined. Premenopausal OEI-positive ovaries showed slightly greater proportion of BRCA1 immunoreactivity (4/13, 31%) than postmenopausal ovaries (4/17, 24%; Table 2). Same was the average percentage of the stained cells (0.54 and 0.47%). BRCA1 expression was present more often in the proliferative phase (3/8, 38%) than in the secretory phase (1/5, 20%; Table 2), with the average percentage of stained cells being 0.75 and 0.20%, respectively. Again these differences were based on small numbers and none are statistically significant. Representative pictures of BRCA1 staining are shown as Fig. 1 (a and c). Morphologically, some BRCA1-positive cells tended to show mild nuclear atypia (Fig. 1a).

To examine whether BRCA1 expression is associated with MIB-1 expression in OEI cells, we used the dual staining method. We showed that all BRCA1 positive cells were positive for MIB-1 immunoreactivity (orange color). Representative pictures of dual staining are shown as Fig. 1 (b and d). Some MIB-1 immunoreactivity was also observed in BRCA1-negative cells. A representative picture with positive MIB-1 and negative BRCA1 is shown in Fig. 1d.

No BRCA1 and/or MIB-1 expression was detected in adjacent stroma in any of these 30 ovaries. OSE was identified in 12 of the 30 OEI-containing ovaries. Two (17%) of the 12 OSE samples showed occasional dual BRCA1 and MIB-1 staining, which was less than observed in OEIs. Again the difference was not statistically significant.

**Fig. 2:**
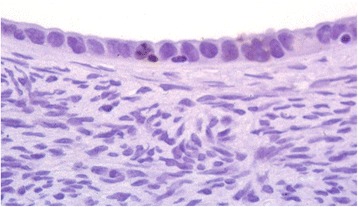
Apoptosis in ovarian epithelial inclusions detected by TUNEL assay. Representative positive apoptotic cell is shown by *dark brown color*. Nuclear fragmentation is apparent. No apoptosis is observed in stromal cells. (Original magnifications × 200)

Apoptotic activity was found in OEI cells from 21 (70%) of the 30 OEI-containing ovaries (Table 3). The apoptotic activity in OEI-containing ovaries was higher in postmenopausal (15/17, 88%) than in pre-menopausal (6/13, 46%) women (*p* < 0.05). This activity also presented more often in the secretory phase (4/5, 80%) than in the proliferative phase (2/8, 25%) (*p* < 0.05). The average percentage of apoptotic cells was lower than 1% of total OEI cells. By examining the parallel sections from dual staining slides, apoptotic cells detected from OEIs were located in different positions from those either BRCA1 and/or MIB-1 positive cells. Representative picture of the OEI apoptosis is shown in Fig. 2.

**Table 3:** Apoptotic activity in OEI cells with certain menopausal status and menstrual cycle phase

	Apoptosis		
	Negative	Positive (%)	
Premenopausal	7	6 (46%)	*p* = 0.02
Postmenopausal	2	15 (88%)	
Total	9	21 (70%)	
Proliferative phase	6	2 (25%)	*p* = 0.03
Secretory phase	1	4 (80%)	
Total	7	6 (46%)

## Discussion

OEIs are frequently seen in the ovarian cortex of both premenopausal and postmenopausal women. In this study, we examined two sections per ovary and observed OEIs in 53% of women examined, which is similar to previous findings [31]. It has been reported that the incidence of OEIs increases with age [31, 32]. In this study, OEIs were more common in the ovaries of postmenopausal women (58%) than of premenopausal women (45%), but there was no trend of increasing numbers of OEIs with age in premenopausal women. The increase of OEI in postmenopausal women was due to the greater number of OEIs in younger postmenopausal women. This pattern is consistent with the results presented by Mittal et al. [10] and Westhoff et al. [12]. Although it needs to be confirmed in a larger study with systematically collected data on the menstrual and reproductive history of women, this pattern suggests that the OEI number is not influenced by the number of ovulation based on this study, but seems increased in the immediate postmenopausal period (probably followed by regression). However, this pattern is not in harmony with the known epidemiologic observations of OEC [8]. The strong correlation we observed for the presence (and number) of OEI in paired ovaries has been previously reported [12], which is compatible with the tubal origin of OEIs instead of coming from ovarian surface epithelia [17, 19, 20].

Previous study of OEIs reported that no mitoses had been observed [10]. This is the first study to investigate epithelial cell proliferation within OEIs by an immunohistochemical method. From the IHC results, we found that 0.4 to 0.9% of OEIs were not in the G_0_ phase of cell cycle. Although the proportion of proliferative OEI cell is low, the pattern is similar to what has been observed in normal breast duct epithelium [33] and it is compatible with the overall incidence of ovarian epithelial cancer of 0.5 to 0.8% [34]. These observations suggest that OEI cells do not invariably disappear, but some of them have a potential of growth. However, it is currently unknown what factors control the growth of OEIs.

In parallel with cell growth, cell death is another important parameter for tumor development. In contrast to proliferative activity in ovarian OEI cells, apoptosis in the ovaries of postmenopausal women was significantly more than that in premenopausal women. Although the overall number of apoptotic cells was small, the observed higher apoptotic activity in postmenopausal (low estrogen level) women supports the well-known epidemiologic phenomenon that the rate of increase in incidence of OECs is much lower in the postmenopausal period than in the premenopausal period [8]. In addition to low estrogen level, high progesterone level (secretory or luteal phase of menstrual cycle) may promote OEI cell death via induction of apoptosis. Significantly higher apoptotic activity within OEIs in secretory phase suggests that intra-ovarian progesterone may have an inhibitory role in OEI development. This coincides with a previous finding of the progesterone-induced dose-response inhibitory effect in ovarian cystadenoma cells in vitro [35]. The inhibitory role of progesterone makes better sense for OEIs, since OEIs are mostly embedded in ovarian stroma and are undoubtedly exposed to high levels of progesterone during ovulation. This may partially explain the epidemiologic observation that pregnancy and oral contraceptive use are protective for OEC [2]. However, the molecular mechanisms for progesterone-related apoptosis in the secretory menstrual phase remain to be defined.

BRCA1 immunoreactivity was seen about half of the time in OEI cells as was MIB-1. All BRCA1 positive cells showed MIB-1 immunoreactivity. This is in agreement with the previous findings in mouse ovary [36], which suggests that BRCA1 is expressed in a cell-cycle-dependent fashion. The expression of BRCA1 mRNA and protein during different stages of cell cycle is highly specific, occurring late in the G1 phase and peeking in the S-phase [37]. BRCA1 expression appears to be a consequence of cell division. This is supported by the observation that BRCA1-immunoreactive cells are positive for MIB-1 expression, which is consistent with the finding that BRCA1 is expressed in proliferating tissues that are not hormonally regulated such as liver [38]. Further studies are needed to determine whether this BRCA1 associated cell proliferation is indirectly related to hormonal status, such as effects from autocrine and paracrine growth factors elaborated by adjacent stromal cells. In our previous study of BRCA1 and MIB-1 expression in ovarian epithelial tumors, we found that BRCA1 was highly correlated with MIB-1 expression in cystadenomas and borderline tumors [25]. If we believe that ovarian epithelial tumorigenesis is a multi-step process, our finding that high correlation of BRCA1 and MIB-1 in OEIs supports the assumption that ovarian epithelial tumors at least some of them may arise from OEIs. Although small percentage (<1%) of the cells were seemingly difficult to explain all existing OEC developing theories, considering the fact that OEC development is a relatively long process, minor imbalance between cell proliferation and cell death in OEIs under the influence of hormonal status may, in a long term run, play an important role in the process of ovarian epithelial tumorigenesis.

It is biologically known that BRCA1 acts in concert with DNA repair enzymes to maintain the integrity of the genome during cell growth [39, 40]. Some BRCA1-positive cells we observed showed mild nuclear atypia. This is likely to be the same phenomenon observed in Werness’ report [16], in which they studied OEIs in ovaries removed prophylactically from women with a family history of ovarian cancer and found more irregular outlines of the epithelial nuclei compared to the control group. These irregular nuclear outlines appear to be equivalent to our ‘mild nuclear atypia’. However, whether DNA damage is present in these mild atypical cells remains to be defined. BRCA1 immunoreactivity in normal ovary was concentrated in the nuclei of granulosa and theca interna cells of developing follicles, the vast majority of which were BRCA1 positive; this is consistent with observations made on mouse ovary [36]. BRCA1 immunoreractivity was also mainly observed in the nuclei of OEI cells. We did not detect BRCA1 immunoreactivity in the stroma of normal ovaries. The non-detectable or rare immunoreactivity of BRCA1 in ovarian stroma is possibly due to the mitotically almost inactive nature of this tissue. This finding is in contrast to that of Wilson et al. [29] who reported that BRCA1 was expressed uniformly in all types of ovarian cells. Further studies are needed to clarify these discrepant findings.

## Conclusions

In summary, this is the first study on BRCA1 expression, cellular proliferation and apoptosis in OEIs of patients without known history of ovarian cancer. BRCA1 expression is closely related to cellular proliferation in OEI cells. Apoptosis of OEI cells is found more in secretory phase of the menstrual cycle and in postmenopausal status. Imbalance between cell proliferation and death within OEI under the influence of hormones may be important for OEC development. Further studies are needed to identify the factors for maintaining their survival and promoting their growth.

## Abbreviations

IHC: Immunohistochemistry
MCF-7 cells: Permanent cell line derived from human breast carcinoma
MIB-1: Proteins expressed in proliferating cells
OC: Oral contraceptive
OEC: Ovarian epithelial cancer
OEI: Ovarian epithelial inclusion, which is mainly derived from fallopian tubal epithelia
OSE: Ovarian surface epithelium
TUNEL: Terminal deoxynucleotidyl transferase-mediated deoxyuridine triphosphate nick end labeling. This is the assay commonly used to identify apoptotic cells in situ
